# In Vitro–In Vivo Correlations (IVIVC) for Predicting the Clinical Performance of Metronidazole Topical Creams Intended for Local Action

**DOI:** 10.3390/pharmaceutics15010268

**Published:** 2023-01-12

**Authors:** Seeprarani Rath, Isadore Kanfer

**Affiliations:** 1 Biopharmaceutics Research Institute, Rhodes University, Grahamstown 6139, South Africa; 2 Leslie Dan College of Pharmacy, University of Toronto, Toronto, ON M5S 3M2, Canada

**Keywords:** in vitro release testing (IVRT), tape stripping, dermatopharmacokinetics (DPK), in vitro–in vivo correlation (IVIVC), bioavailability/bioequivalence (BA/BE), apparent release constants (ARCs), area under the curve (AUC)

## Abstract

The safety and efficacy of a generic medicine can be confirmed by demonstrating bioequivalence (BE) between the generic product and its reference listed drug (RLD) by measuring drug concentrations in the blood following administration. However, for topical dermatological products that are not absorbed into the systemic circulation, clinical trials in patients are required. The objective of this investigation was to use an in vitro method to predict in vivo performance by correlating in vitro release testing (IVRT) data with tape stripping (TS) data following the application of metronidazole (MTZ) creams to the skin of healthy human participants. Whereas IVRT is generally used to characterize the release of a drug from topical products across a synthetic membrane into a suitable receptor medium, TS involves the sequential removal of layers of stratum corneum (SC) with an adhesive tape to determine the amount of the drug in the skin. The resulting IVRT and TS data were correlated using the IVRT parameter of the apparent release constant (ARC), which is the slope obtained from the release rate profile, with the TS parameter of the area under the curve (AUC) obtained from a plot of the amount of drug per tape strip vs. the relative SC depth. A rank order relationship for these parameters was established for the reference and test products. A graph of AUC vs. ARC was plotted to establish a Level C in vitro–in vivo correlation (IVIVC). Although the ARC for T_1_ was slightly lower than that for the reference, the rank order was essentially consistent. A linear relationship was observed between the AUCs and ARCs. The equation derived was used to predict the AUCs for all the tested products based on their respective ARCs. The predicted AUC values based on the observed ARCs were similar to the observed AUCs. The lower and upper limits for the in vitro and in vivo parameters for BE were computed based on regulatory acceptance criteria. In order to predict BE from the IVRT studies, the values of the ARC should be between 30.50 and 47.67 when comparing test and reference cream products containing MTZ.

## 1. Introduction

The United States Food and Drug Administration (FDA) defines in vitro–in vivo correlation (IVIVC) as “a predictive mathematical model describing the relationship between an in vitro property of a dosage form and a relevant in vivo response” [1]. The FDA published regulatory guidance on the development, evaluation, and application of IVIVC for extended-release (ER) oral dosage forms in 1997 [1]. IVIVC has been one of the most important issues in the field of pharmaceutics in attempts to develop in vitro systems and models for predicting the clinical performance of drug products. Although no regulatory IVIVC guidance for topical drug products has been published yet, the principles described in the FDA’s IVIVC Guidance for ER oral dosage forms have been applied to develop IVIVC for such products [2]. In vitro parameters such as the drug release from the dosage forms obtained from in vitro release testing (IVRT) or steady-state flux, diffusivity, and partition coefficient obtained from in vitro permeation testing (IVPT) or rheological properties (e.g., viscosity, spreadability, etc.) and in vivo parameters such as the amount of the drug in the *stratum corneum* (SC) obtained from tape stripping (TS), (i.e., dermatopharmacokinetic (DPK) studies) or the area under the effect curve (AUEC) obtained from the vasoconstrictor assay (VCA) (i.e., pharmacodynamic (PD) studies) may be used to establish an IVIVC of topically applied drug products [3].

The FDA’s IVIVC guidance for ER oral dosage forms defines four levels of correlation, as described below [2].

- Level A represents a point-to-point relationship between in vitro and in vivo profiles. Although these correlations are generally linear, non-linear correlations are also acceptable. It is considered to be the most informative and is the only level of IVIVC accepted by the FDA in obtaining biowaiver.
- Level B correlation utilizes the principles of statistical moment analysis. A mean in vitro dissolution time (MDT_in vitro_) is compared to either a mean in vivo residence (MRT_in vivo_) or dissolution time (MDT_in vivo_). However, since various in vivo release profiles may result in the same MRT_in vivo_ or MDT_in vivo_, Level B correlation is not considered to be a point-to-point correlation. Additionally, this kind of correlation does not necessarily reflect the actual in vivo plasma profile, which may result in insufficient predictability.
- Level C involves a single-point correlation between a dissolution parameter (e.g., T_50%_, T_90%_) and a pharmacokinetic parameter such as peak plasma concentration (C_max_), time to reach C_max_ (T_max_), or area under the curve (AUC). However, it may not be adequate in predicting in vivo drug performance because single-point analysis does not appropriately reflect the complete shape of the plasma concentration time-curve, which is critical to explain the in vivo performance of the formulation. Nevertheless, such correlations may be employed in the early stages of formulation development while selecting pilot formulations.
- Multiple Level C correlation relates multiple dissolution time points to one or more pharmacokinetic parameter(s) (e.g., C_max_, T_max_, or AUC). At least three dissolution time points covering the early, middle, and late stages of the dissolution profile should be included to establish such a correlation. A multiple Level C correlation can be as useful as a Level A correlation. Furthermore, the development of a multiple Level C correlation also indicates that establishing a more preferable Level A correlation is feasible.

Additionally, a rank order correlation comparing in vitro and in vivo release profiles (Level D) can also be established. It serves as an aid in selecting the formulation and process variables during the early stages of product development. However, since this type of correlation only provides qualitative information, it is not included in the FDA’s IVIVC Guidance [2,4].

### Applications of IVIVC

A biowaiver can be obtained, whereby an in vitro release method can be used as a surrogate for BE studies for pre-approval and post-approval changes to the approved product if a Level A correlation has been established and validated. A successfully developed IVIVC may be used to accurately predict the in vivo performance of a drug product from its in vitro performance, circumventing the need for in vivo studies and thereby lowering the economic and regulatory burden. Biowaivers facilitate the faster development of generic products, whereby BE between generic and innovator products can be expedited. Additionally, a meaningful IVIVC can be used as a guide in both formulation and process development during the various stages of drug product development. It can also be used to support and validate the use of an in vitro method and to control clinically relevant dissolution parameters to ensure the safety and efficacy of the final product [2].

Although obtaining an IVIVC for topical products is challenging, some studies have shown promising results. Most of these studies demonstrate a relationship between in vitro drug release and PD and/or DPK measurements [3].

Herkenne et al. [5] assessed the transport of ibuprofen into the skin from a topical formulation by comparing various in vitro (IVPT) and in vivo (TS) parameters such as the steady-state flux, diffusivity and partition coefficient. Although in vitro drug release was performed using both silicone membranes (IVRT) and excised porcine ear skin (IVPT), IVPT was considered for comparisons since the fluxes obtained using IVRT were not in complete agreement with those obtained from IVPT or the behavior of the formulations in vivo. A rank order relationship was established between the in vitro (IVPT) and in vivo (TS) parameters, which demonstrated a good correlation. In a study reported by Caron et al. [6], two topical hydrocortisone formulations were compared using VCA (PD), TS (DPK), and IVRT. A good rank order correlation was observed between the in vitro (IVRT) and in vivo (PD and DPK) responses, i.e., the amount released in 6 h (μg/cm^2^), vasoconstriction at 16 h, and hydrocortisone concentration in the skin (μg/cm^2^). A good rank order relationship between the in vivo skin blanching response and in vitro release rate of betamethasone valerate from topical creams was observed in a study published by Shah and co-workers [7]. In a study conducted by Cordery et al. [8], the in vitro and in vivo fluxes of topical diclofenac formulations following IVPT using porcine skin and TS in human participants, respectively, showed good qualitative and quantitative correlations. The DPK of terbinafine hydrochloride topical formulations was assessed across excised pig ear skin and in healthy human participants using TS, and a relationship between them was established by Saeheng et al. [9]. The partition coefficient and diffusion parameter from the in vitro TS were used to predict the in vivo AUC using pharmacokinetic (PK) software. Welin-Berger et al. [10] established a good correlation between the in vivo and in vitro fluxes of a topically applied local anaesthetic agent. The in vitro permeation was studied across excised human skin using static diffusion cells, whereas in vivo studies involved the withdrawal of blood samples following topical application. In a proof-of-concept study, Mohammed et al. [11] investigated the permeation of niacinamide in vitro through excised human skin using Franz diffusion cells and in vivo through healthy human skin using Confocal Raman Spectroscopy (CRS). A good correlation was observed between the in vitro flux of the drug and the signal intensity of the drug at a depth of 4 μm in the SC. Similarly, Mateus et al. [12] studied the permeation of salicylic acid in vitro across excised porcine ear skin and in vivo in healthy human volunteers using CRS. The cumulative amount of drug that permeated per unit area of porcine skin and the amount of drug that permeated in vivo through the SC at a depth of 2 μm demonstrated a good correlation.

Apart from the FDA’s VCA for products containing topical corticosteroids, most regulatory agencies require clinical endpoint studies in patients to obtain market approval for topical products intended for local action. These expensive and arduous requirements have resulted in a dearth of such products, where topical generic products containing metronidazole (MTZ) are conspicuously absent in many countries. Topical formulations containing MTZ are widely used to treat inflammatory lesions of rosacea. The objective of this investigation was to use an in vitro method to predict in vivo performance by correlating in vitro release testing (IVRT) data with tape stripping (TS) data following the application of MTZ creams to the skin of healthy human participants. In order to establish a good IVIVC, three products with different in vitro and in vivo measurements were used.

## 2. Materials and Methods

### 2.1. Materials

Metrocreme^®^ (Galderma Laboratorium GmbH, Düsseldorf, Germany) containing 0.75% MTZ was used as the reference MTZ cream, and specially manufactured creams containing an equal strength (0.75%), 25% less (0.56%), and 26% more (0.95%) MTZ compared to the reference product were used as test products: T_1_, T_2_, and T_3_, respectively.

### 2.2. Method

Comparative IVRT studies were performed as previously described [13] using the reference, Metrocreme^®^, 0.75% MTZ, and test creams T_1_, T_2_, and T_3_ containing 0.75%, 0.56%, and 0.95% MTZ. For practical reasons, two separate sets of IVRT runs were performed using the same reference, where T_1_ was compared to the reference in run 1 and T_2_ and T_3_ were compared to the reference in run 2. A Hanson VDC system (Hanson Research Corporation, Chatsworth, CA, USA) equipped with six vertical diffusion cells (Volume: 7.9 mL, orifice: 15 mm) was used for the IVRT studies. The donor and receptor chambers of the VDCs were separated using a synthetic membrane, Magna Nylon (0.45 µm, 25 mm, GVS Life Sciences, Sanford, ME, USA). SABAX Pour Saline (0.9% NaCl solution, Adcock Ingram Critical Care (Pty) Ltd., Midrand, South Africa) served as the receptor medium. The receptor medium was continuously stirred, and the temperature was maintained at 32 °C to mimic the physiological temperature. Two hundred microliter samples were withdrawn at 15 min intervals for 90 min, and the VDCs were replenished with equivalent volumes of the receptor medium. The samples were analyzed using a Waters Acquity Ultra-Performance Liquid Chromatography (UPLC) system (Waters, Milford, MA, USA) [13].

The DPK of the above-mentioned formulations were assessed using the TS methodology described in another publication from our laboratory [14]. All the products were assessed simultaneously in the TS study using ten healthy participants (4 males, 6 females) between the ages of 23 and 31 (mean 27 years). The volar aspects of each of the forearms of each participant were demarcated to provide five sampling sites (2 × 2 cm). Four of the sites were used for product application (reference and test products, T_1_, T_2_ and T_3_), and one site on each arm was assigned as a blank. Approximately 15 mg of each cream were applied to each application site. The creams were removed after an application time of 60 min, and each site was sequentially stripped using 20 pre-weighed tape strips (Scotch^TM^ Magic Tape, 3M, Johannesburg, South Africa). The amount of SC removed was determined by re-weighing the individual tape strips immediately after stripping. All application sites were randomized between participants. An UPLC method was used to determine the amount of MTZ in the relevant tape strips. The thickness of the SC on each participant was determined from transepidermal water loss (TEWL) measurements conducted on the blank site. One treatment each of the reference and the three test products were applied on each arm of every participant [14].

The release rates obtained from the slopes of plots of cumulative amount of drug released per unit area against the square root of time in minutes are referred to as apparent release constants (ARCs). The resulting IVRT and TS data were correlated using the IVRT parameter, ARC, with the TS parameter, area under the curve (AUC), which is obtained from a plot of the amount of drug per tape strip vs. relative SC depth [15,16]. A rank order relationship was established using the release rates and AUCs observed for the reference and test products during the IVRT [13] and in vivo TS studies [14], respectively.

Furthermore, the relevant ARCs were plotted against the corresponding AUCs. The resulting data yielded a linear relationship, and the equation obtained from this plot was then used to predict the AUCs for all the tested products based on their respective ARCs. In addition, BE limits (0.8–1.25) for these in vitro and in vivo parameters, i.e., ARC and AUC, respectively, were computed based on a regulatory acceptance criterion of a ≤20% difference between the test and reference products.

## 3. Results

**Figure 1:**
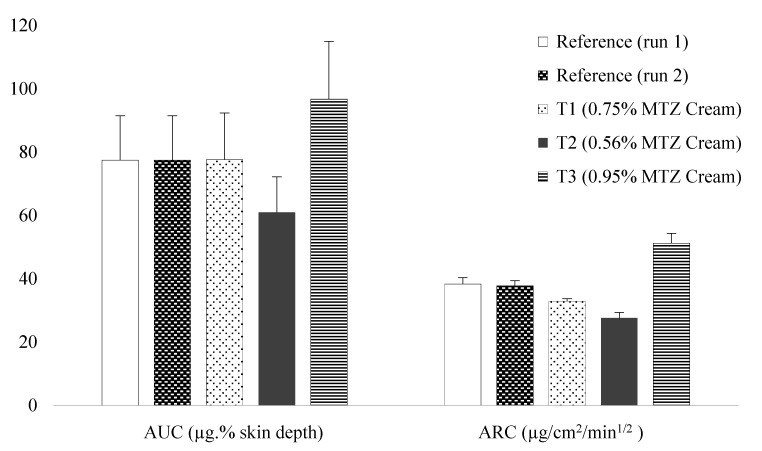
Comparison of AUC and ARC deduced from the TS study (in vivo) in human participants (n = 10) and IVRT studies (in vitro) (n = 6).

Figure 1 depicts a rank order relationship between the in vitro and in vivo measurements. Although the histogram shows a slightly lower ARC for T_1_, the rank order is essentially consistent with the strengths. It can be clearly seen that the bars for reference creams and T_1_ are similar, whereas those for T_2_ are lower than and those for T_3_ are higher than the reference, correlating with the lower and higher strengths, respectively. The IVRT and TS data are provided as Supplementary Materials (Tables S1–S4).

**Figure 2:**
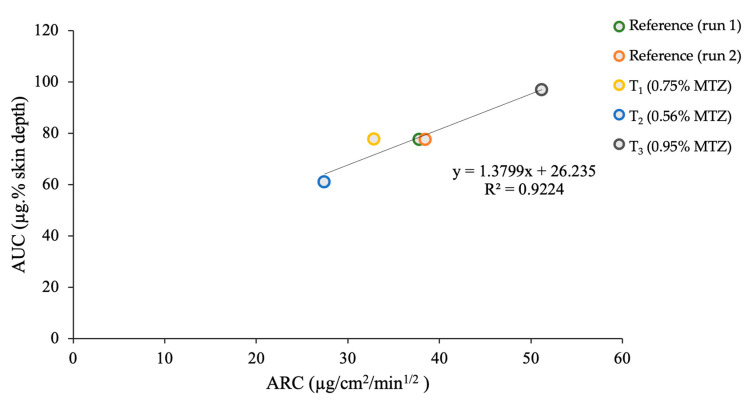
Relationship between the AUC and ARC deduced from the TS study (in vivo) in human participants (n = 10) and IVRT studies (in vitro) (n = 6).

Based on the linear relationship (R^2^ > 0.9) between the in vitro and in vivo parameters (Figure 2), the resulting equation, y = 1.3799x + 26.235, was used to predict the AUCs based on the ARCs, and the values obtained are depicted in Table 1.

The predicted AUC values were similar to the observed values (CV < 10%). The lower and upper limits for the in vitro and in vivo parameters for BE are provided in Table 2.

Based on the above data, it can be seen that in order to show BE using in vitro studies, the values of the ARC need to be between 30.50 and 47.67 µg/cm^2^/min^1/2^ when comparing the test and reference cream products containing MTZ.

## 4. Discussion

The rank order relationships of in vitro and in vivo parameters—ARC and AUC, respectively—for the various formulations were in agreement with the strengths of each relevant MTZ formulation. A meaningful Level C correlation between the in vitro (ARC) and in vivo (AUC) parameters was established using the reference and three test products, as suggested in the FDA’s IVIVC Guidance. The observed AUC values for the reference and T_1_ (0.75% MTZ) products were well within the acceptance limits of BE (0.8–1.25). The closeness of the observed AUC values to the predicted AUC values indicated the potential of the developed IVIVC to predict in vivo behavior based on IVRT results. Furthermore, it can be used as a foundation to establish additional IVIVCs, which can pave the way for obtaining a biowaiver for topical MTZ cream products and possibly also other topical products using both IVRT and TS data. A multiple Level C correlation can be established using parameters such as the cumulative amounts released at various time points during the IVRT run versus AUC or the total amount penetrated in vivo, whereas a Level A correlation can also be obtained using a point-to-point relationship.

## 5. Conclusions

An IVIVC was successfully developed based on the ARCs obtained from the IVRT studies correlated with the AUC data obtained from the TS studies. Using these correlations, predictions on possible clinical performance can be obtained from the data generated from IVRT studies, which correspond to the in vivo data indicated by the AUC values from TS studies. The resulting data indicated that T_1_ can be considered BE to the reference since the ARC value was 32.89 ± 1.60 µg/cm^2^/min^1/2^, which fell within the range of 30.50 to 47.67 µg/cm^2^/min^1/2^, and the AUC fell within the range of 68.33 to 92.02 µg.% skin depth, which corresponded to the BE acceptance limits of 0.8–1.25. However, T_2_ and T_3_ are considered to be non-BE since the ARC values, 27.42 ± 0.99 and 51.20 ± 0.60 µg/cm^2^/min^1/2^, corresponded to predicted AUC values of 64.07 and 96.89 µg.% skin depth, respectively, which fell outside the BE acceptance limits. The results reveal that IVRT data can be used to predict BE of MTZ topical creams following the successful development of an appropriate IVIVC.

## Figures and Tables

**Table 1:** Predicted AUC values obtained from ARC values.

Product	AUC (µg.% Skin Depth)	ARC (µg/cm^2^/min^1/2^)	Predicted AUC (µg.% Skin Depth)
90% CI * (0.95–1.06)			
Reference (run 1)	77.46 ± 13.48	37.80 ± 2.01	78.40
T_1_ (0.75% MTZ)	77.62 ± 13.75	32.89 ± 1.60	71.62
90% CI * (0.74–0.84)			
Reference (run 2)	77.46 ± 13.48	38.47 ± 1.35	79.32
T_2_ (0.56% MTZ)	60.98 ± 10.30	27.42 ± 0.99	64.07
90% CI * (1.20–1.30)			
Reference (run 2)	77.46 ± 14.01	38.47 ± 1.35	79.32
T_3_ (0.95% MTZ)	96.78 ± 15.77	51.20 ± 0.60	96.89

* Confidence interval.

**Table 2:** BE limits for AUC and ARC.

BE Limits	Predicted AUC (µg.% Skin Depth)	ARC (µg/cm^2^/min^1/2^)
0.7999 (lower)	68.33	30.50
1.2501 (upper)	92.02	47.67

## Data Availability

Data will be provided on request.

## Supplementary Materials

The following supporting information can be downloaded at: https://www.mdpi.com/article/10.3390/pharmaceutics15010268/s1. Table S1: 0.75% MTZ cream vs. Metrocreme^®^, 0.75% MTZ (IVRT run 1); Table S2: 0.56% MTZ cizzy.kanfer@utoronto.caream vs. Metrocreme^®^, 0.75% MTZ (IVRT run 2); and Table S3: 0.95% MTZ cream vs. Metrocreme^®^, 0.75% MTZ (IVRT run 2); Table S4: AUC values for the reference and test products for each of the ten participants.
