# Supplementary material for: In Vitro–In Vivo Correlations (IVIVC) for Predicting the Clinical Performance of Metronidazole Topical Creams Intended for Local Action
Source: Pharmaceutics. 2023 Jan 12;15(1):268. doi: 10.3390/pharmaceutics15010268 (PMC9863435; doi:10.3390/pharmaceutics15010268)
Supplement: Supplementary file 1 [file pharmaceutics-15-00268-s001.zip › pharmaceutics-2114687-supplementary.pdf]

The following tables contain raw data for the results presented in Table 1 of the manuscript:

A] The release rates/ apparent release constants (ARCs) obtained from IVRT studies using six vertical diffusion cells (VDCs) for the following products –

Table S1. 0.75% MTZ cream vs Metrocreme®, 0.75% MTZ (IVRT run 1)

Product	ARC values (µg/cm^2/min$^{1/2}$)						
	**VDC 1**	**VDC 2**	**VDC 3**	**VDC 4**	**VDC 5**	**VDC 6**	**Mean ± SD**
0.75% MTZ Cream (T_1)	32.05	31.51	32.97	32.68	35.99	32.16	32.89 ± 1.60
Metrocreme®, 0.75% MTZ (Reference – run 1)	38.56	40.09	35.82	36.99	35.47	39.88	37.80 ± 2.01

Table S2. 0.56% MTZ cream vs Metrocreme®, 0.75% MTZ (IVRT run 2)

Product	ARC values (µg/cm^2/min$^{1/2}$)						
	**VDC 1**	**VDC 2**	**VDC 3**	**VDC 4**	**VDC 5**	**VDC 6**	**Mean ± SD**
0.56% MTZ cream (T_2)	28.30	25.87	27.38	28.66	26.98	27.34	27.42 ± 0.99
Metrocreme®, 0.75% MTZ (Reference – run 2)	37.13	40.59	37.91	37.46	38.14	39.62	38.47 ± 1.35

Table S3. 0.95% MTZ cream vs Metrocreme®, 0.75% MTZ (IVRT run 2)

Product	ARC values (µg/cm^2/min$^{1/2}$)						
	**VDC 1**	**VDC 2**	**VDC 3**	**VDC 4**	**VDC 5**	**VDC 6**	**Mean ± SD**
0.95% MTZ cream (T_3)	50.24	51.98	51.54	51.41	50.86	51.17	51.20 ± 0.60
Metrocreme®, 0.75% MTZ (Reference – run 2)	37.13	40.59	37.91	37.46	38.14	39.62	38.47 ± 1.35

B] Area under the curve (AUC) values obtained following tape stripping (TS) studies

Table S4. AUC values for the reference and test products for each of the ten participants

Participant ID	AUC values (µg.% skin depth)			
	Metrocreme®, 0.75% MTZ (Reference)	0.75% MTZ cream (T_1)	0.56% MTZ cream (T_2)	0.95% MTZ cream (T_3)
001	60.66	63.94	46.72	80.17
002	83.92	93.74	60.24	121.73
003	63.29	58.67	49.01	74.41
004	92.90	73.48	62.75	97.12
005	72.37	75.12	53.65	87.60
006	82.46	90.68	68.49	95.84
007	76.21	76.33	71.01	101.83
008	99.07	95.56	70.10	115.56
009	59.80	61.01	51.79	83.09
010	83.91	87.70	76.04	110.48
Mean ± SD	77.46 ± 13.48	77.62 ± 13.75	60.98 ± 10.30	96.78 ± 15.77
